# Cancer survival in the United States 2007–2016: Results from the National Program of Cancer Registries

**DOI:** 10.1371/journal.pone.0284051

**Published:** 2023-05-11

**Authors:** Taylor D. Ellington, S. Jane Henley, Reda J. Wilson, Virginia Senkomago, Manxia Wu, Vicki Benard, Lisa C. Richardson

**Affiliations:** 1 Oak Ridge Institute for Science and Education, Oak Ridge, Tennessee, United States of America; 2 Division of Cancer Prevention and Control, National Center for Chronic Disease Prevention and Health Promotion, Centers for Disease Control and Prevention, Atlanta, Georgia, United States of America; University of Pennsylvania, UNITED STATES

## Abstract

**Background:**

Cancer survival has improved for the most common cancers. However, less improvement and lower survival has been observed in some groups perhaps due to differential access to cancer care including prevention, screening, diagnosis, and treatment.

**Methods:**

To further understand contemporary relative cancer survival (one- and five- year), we used survival data from CDC’s National Program of Cancer Registries (NPCR) for cancers diagnosed during 2007–2016. We examined overall relative cancer survival by sex, race and ethnicity, age, and county-level metropolitan and non-metropolitan status. Relative cancer survival by metropolitan and non-metropolitan status was further examined by sex, race and ethnicity, age, and cancer type.

**Results:**

Among persons with cancer diagnosed during 2007–2016 the overall one-year and five-year relative survival was 80.6% and 67.4%, respectively. One-year relative survival for persons living in metropolitan counties was 81.1% and 77.8% among persons living in non-metropolitan counties. We found that persons who lived in non-metropolitan counties had lower survival than those who lived in metropolitan counties, and this difference persisted across sex, race and ethnicity, age, and most cancer types.

**Conclusion:**

Further examination of the differences in cancer survival by cancer type or other characteristics might be helpful for identifying potential interventions, such as programs that target screening and early detection or strategies to improve access to high quality cancer treatment and follow-up care, that could improve long-term outcomes.

**Impact:**

This analysis provided a high-level overview of contemporary cancer survival in the United States.

## Introduction

Even though cancer incidence rates stabilized during 2013 to 2016 among males and increased slightly among females, cancer death rates continued to decline steadily among both males and females [1]. Changes in incidence and mortality reflect, in part, changes in cancer risk factors and screening and imaging test use, while declines in cancer mortality may also be due to improvements in early detection and treatment of cancer. Differences in stage at diagnosis, timeliness of follow-up after diagnosis, appropriate treatment after diagnosis, or having a chronic condition may impact differences in survival after cancer diagnosis [2]. Hence, along with incidence and death rates, examining cancer survival is useful in assessing progress in efforts to improve cancer outcomes [3, 4].

Cancer survival has improved since the mid‐1970s, overall and for several common cancers [4]. However, less improvement and lower survival has been observed in some groups–defined by age, race and ethnicity, geography, and other characteristics–perhaps due to differential access to cancer care including prevention, screening, diagnosis, and treatment. Previous studies have found five-year relative survival is highest when cancer is diagnosed before age 45 years and decreases with increasing age [5]. Differences in survival rates have been observed among racial and ethnic groups. One study found Black cancer patients have lower five-year relative survival compared to cancer patients in other racial and ethnic groups [6]. Many studies, particularly of prostate and colorectal cancer, have observed lower survival among cancer patients who live in rural areas compared to those who live in urban areas [7].

To further the understanding of recent relative cancer survival (one- and five- year), we used survival data from CDC’s National Program of Cancer Registries (NPCR) for cancers diagnosed during 2007–2016. We examined overall relative cancer survival by sex, race and ethnicity, age and county-level metropolitan and non-metropolitan status. Relative cancer survival by metropolitan and non-metropolitan status was further examined by sex, race and ethnicity, age, and cancer type.

## Methods

### Data source

#### Cancer survival data

Data on survival patterns of cancers diagnosed in the United States diagnosed during 2007–2016, the most recently available data, were obtained from the NPCR survival dataset. The data set, which covers 94% of the U.S. population, includes 45 population-based cancer registries that met U.S. Cancer Statistics (USCS) publication criteria and conducted active patient follow-up or linkage with CDC’s National Center for Health Statistics (NCHS) National Death Index to determine vital status [8]. Cancers were coded using *International Classification of Diseases for Oncology*, *Third Edition* [9]. Relative survival was examined by sex, race and ethnicity, age, cancer type, and metropolitan and non-metropolitan status.

#### Race and ethnicity

Central cancer registries gather and collate information about race and Hispanic or Latino ethnicity from self-reported intake questionnaires, abstracted patient records, and linkages to administrative databases. An algorithm was applied to Hispanic ethnicity data to reduce misclassification of Hispanic persons as being of unknown ethnicity [10]. Up to 5 fields were used to record race [11]. Standard algorithms were used to bridge these fields into 4 race groups (White, Black, American Indian or Alaska Native, and Asian or Pacific Islander) to correspond to race data collected on death certificates through 2018 [12]. To reduce misclassification of American Indian or Alaska Native race, central cancer registries link case data according to a certain protocol with the Indian Health Service (IHS) patient registration database. The database contains records of individuals who are members of federally recognized tribes who utilize the IHS/Tribal Health System/Urban Indian Organization healthcare system; cases linked with the IHS database were coded as AI/AN [13]. In this analysis, race and ethnicity data were combined as: non-Hispanic white, non-Hispanic Black, non-Hispanic American Indian or Alaska Native (AI/AN), non-Hispanic Asian or Pacific Islander, and Hispanic/Latino including all races.

#### Population

Age-specific analyses used these grouped categories <50, 50–59, 60–69, 70–79, and ≥80 years.

#### Metropolitan and non-metropolitan status

The U.S. Department of Agriculture Economic Research Service 2013 vintage rural-urban continuum classification scheme was used to categorize county of residence at diagnosis as metropolitan (rural-urban continuum codes 1–3) or non-metropolitan (rural-urban continuum codes 4–9) [14]. We examined relative cancer survival for metropolitan versus non-metropolitan status by sex, race and ethnicity, age, and cancer type.

#### Cancer type

Survival was examined by metropolitan and non-metropolitan status for 10 cancer sites. These cancers were chosen based on the 10 most common sites according to rates of new cancer cases in the United States in 2017. Sites include female breast, prostate, lung and bronchus, colon and rectum, corpus and uterus, melanoma of the skin, urinary bladder, non-Hodgkin lymphoma, kidney and renal pelvis, and leukemia.

### Statistical methods

#### Survival

We calculated one- and five-year relative survival for individuals diagnosed with cancer during 2007–2016 with follow-up through 2016 using SEER*Stat 8.3.8 [15]. One-year and five-year relative survival estimates the percentages of persons who did not die from their cancer within one and five years after cancer diagnosis. Relative cancer survival is a ratio of observed to expected survival and was calculated as the observed all-cause survival in a group of individuals with cancer divided by the expected all-cause survival of a similar group of individuals in the general population [16], assuming that cancer deaths were a negligible proportion of all deaths. Expected survival was based on expected life tables stratified by age, sex, race and ethnicity, socioeconomic status, geography, and year [17, 18]. Relative survival was calculated using the Ederer II actuarial method [16] with the complete analysis approach to include the shorter follow-up time of patients diagnosed in more recent diagnosis years [19]. To allow for informal comparisons, without specifying a referent group, 95% confidence intervals (CIs) for survival estimates are presented. Survival between groups was described as different if 95% CIs did not overlap.

## Results

Among persons with cancer diagnosed during 2007–2016 (N = 12,080,554), the overall one-year and five-year relative survival was 80.6% and 67.4%, respectively (Table 1).

**Table 1:** Overall relative survival cancer survival- United States, 2007–2016^a^.

Cumulative		*n*	Relative	95% lower	95% upper
	One-year	12,080,554	80.6%	80.6%	80.6%
	Two-year	12,080,554	74.5%	74.4%	74.5%
	Three-year	12,080,554	71.1%	71.1%	71.2%
	Four-year	12,080,554	69.0%	68.9%	69.0%
	Five-year	12,080,554	67.4%	67.4%	67.4%
**Sex**					
Male	One-year	6,181,602	79.5%	79.5%	79.5%
	Five-year	6,181,602	66.7%	66.6%	66.7%
Female	One-year	5,898,952	81.8%	81.7%	81.8%
	Five-year	5,898,952	68.2%	68.1%	68.2%
**Race/Ethnicity** ^**b**^					
Non-Hispanic White	One-year	9,172,677	80.5%	80.4%	80.5%
	Five-year	9,172,677	67.5%	67.5%	67.6%
Non-Hispanic Black	One-year	1,376,188	78.4%	78.4%	78.5%
	Five-year	1,376,188	62.7%	62.6%	62.8%
Non-Hispanic American Indian/Alaska Native	One-year	66,854	77.3%	77.0%	77.7%
	Five-year	66,854	62.1%	61.6%	62.5%
Non-Hispanic Asian or Pacific Islander	One-year	363,145	82.5%	82.4%	82.7%
	Five-year	363,145	67.9%	67.7%	68.1%
Hispanic (All Races)	One-year	953,726	82.2%	82.1%	82.3%
	Five-year	953,726	68.6%	68.5%	68.7%
**Age**					
<50 years	One-year	1,851,216	91.9%	91.9%	91.9%
	Five-year	1,851,216	81.0%	80.9%	81.0%
50–59 years	One-year	2,510,522	85.4%	85.3%	85.4%
	Five-year	2,510,522	71.8%	71.7%	71.8%
60–69 years	One-year	3,439,770	82.7%	82.6%	82.7%
	Five-year	3,439,770	69.4%	69.3%	69.5%
70–79 years	One-year	2,641,580	76.2%	76.1%	76.2%
	Five-year	2,641,580	62.1%	62.0%	62.2%
80+ years	One-year	1,637,466	62.9%	62.8%	62.9%
	Five-year	1,637,466	48.2%	48.0%	48.3%

^a^ Data were compiled from 45 population-based cancer registries that participate in the National Program of Cancer registries, meet the data-quality standards for inclusion in U.S. Cancer Statistics, and meet the criteria for inclusion in the survival data set, which covers approximately 94% of the U.S. population.

^b^ Racial and ethnic groups are mutually exclusive. Hispanic persons can be any race.

One-year relative survival was 79.5% among males and 81.8% among females. Relative survival from one to five years decreased 12.8 percentage points (79.5% to 66.7%) among males and 13.6 percentage points (81.8% to 68.2%) among females.

One-year relative survival differed by race and ethnicity: 82.5% for non-Hispanic Asian or Pacific Islander persons, 82.2% for Hispanic persons, 80.5% for non-Hispanic White persons, 78.4% for non-Hispanic Black persons, and 77.3% for non-Hispanic American Indian or Alaska Native persons. Relative survival from one to five years decreased 15.7 percentage points among non-Hispanic Black persons (78.4% to 62.7%), 15.2 among non-Hispanic American Indian/Alaska Native persons (77.3% to 62.1%), 14.6 among non-Hispanic Asian or Pacific Islander persons (82.5% to 67.9%), 13.6 among Hispanic persons (82.2% to 68.6%) and 13.0 among non-Hispanic White persons (80.5% to 67.5%).

One-year relative survival declined with increasing age: 91.9% among persons 50 years of age or younger, 85.4% among persons aged 50–59 years, 82.7% among persons aged 60–69 years, 76.2% among persons aged 70–79 years, and 62.9% among persons aged 80 years or older. Relative survival from one to five years decreased 10.9 percentage points among persons 50 years of age or younger, 13.6 percentage points among persons aged 50–59 years, 13.3 percentage points among persons aged 60–69 years, 14.1 percentage points among persons aged 70–79 years, and 14.7 percentage points among persons aged 80 years or older.

### Metropolitan and non-metropolitan status

One-year relative survival was 81.1% for persons living in metropolitan counties and 77.8% among persons living in non-metropolitan counties. Relative survival from one to five years decreased 13.0 percentage points (81.1% to 68.1%) among persons living in metropolitan counties and 14.2 percentage points (77.8% to 63.6%) among persons living in non-metropolitan counties (Table 2).

**Table 2:** Relative survival by metropolitan and non-metropolitan status^a^ by sex, race/ethnicity^b^, and age- United States, 2007–2016^c^.

		Metropolitan				non-Metropolitan			
		*n*	Relative	95% lower	95% upper	*n*	Relative	95% lower	95% upper
**Total**	One-year	10,114,432	81.1%	81.1%	81.2%	1,959,573	77.8%	77.8%	77.9%
	Five-year	10,114,432	68.1%	68.1%	68.2%	1,959,573	63.6%	63.5%	63.7%
**Sex**									
Male	One-year	5,134,339	80.1%	80.0%	80.1%	1,043,276	76.6%	76.5%	76.7%
	Five-year	5,134,339	67.5%	67.4%	67.5%	1,043,276	62.6%	62.5%	62.8%
Female	One-year	4,980,093	82.2%	82.2%	82.3%	916,297	79.3%	79.2%	79.4%
	Five-year	4,980,093	68.8%	68.7%	68.9%	916,297	64.6%	64.5%	64.7%
**Race/Ethnicity**									
Non-Hispanic White	One-year	7,456,195	81.1%	81.0%	81.1%	1,713,027	77.9%	77.9%	78.0%
	Five-year	7,456,195	68.4%	68.3%	68.4%	1,713,027	63.8%	63.7%	63.9%
Non-Hispanic Black	One-year	1,228,955	78.8%	78.7%	78.9%	146,570	75.4%	75.2%	75.7%
	Five-year	1,228,955	63.2%	63.1%	63.3%	146,570	58.3%	57.9%	58.6%
Non-Hispanic American Indian/Alaska Native	One-year	38,277	78.7%	78.3%	79.2%	28,507	75.4%	74.8%	75.9%
	Five-year	38,277	63.8%	63.1%	64.4%	28,507	59.7%	59.0%	60.5%
Non-Hispanic Asian or Pacific Islander	One-year	356,441	82.6%	82.5%	82.7%	6,642	79.5%	78.5%	80.5%
	Five-year	356,441	68.0%	67.8%	68.2%	6,642	63.2%	61.7%	64.6%
Hispanic (All Races)	One-year	903,683	82.4%	82.3%	82.5%	49,841	78.7%	78.3%	79.1%
	Five-year	903,683	68.9%	68.7%	69.0%	49,841	63.6%	63.0%	64.1%
**Age**									
<50 years	One-year	1,606,470	92.2%	92.1%	92.2%	243,771	89.9%	89.8%	90.0%
	Five-year	1,606,470	81.5%	81.4%	81.6%	243,771	77.5%	77.3%	77.7%
50–59 years	One-year	2,123,080	85.9%	85.8%	85.9%	386,192	82.5%	82.4%	82.6%
	Five-year	2,123,080	72.6%	72.5%	72.6%	386,192	67.4%	67.3%	67.6%
60–69 years	One-year	2,853,135	83.2%	83.1%	83.2%	584,714	80.1%	80.0%	80.2%
	Five-year	2,853,135	70.2%	70.1%	70.2%	584,714	65.7%	65.5%	65.8%
70–79 years	One-year	2,165,848	76.7%	76.6%	76.7%	474,243	73.9%	73.8%	74.0%
	Five-year	2,165,848	62.7%	62.6%	62.8%	474,243	59.3%	59.1%	59.5%
80+ years	One-year	1,365,899	63.1%	63.0%	63.1%	270,653	61.8%	61.6%	62.0%
	Five-year	1,365,899	48.4%	48.2%	48.5%	270,653	47.0%	46.7%	47.4%

^a^ The U.S. Department of Agriculture Economic Research Service 2013 vintage rural-urban continuum classification scheme was used to categorize county of residence at diagnosis as metropolitan (rural-urban continuum codes 1–3) or non-metropolitan (rural-urban continuum codes 4–9).

^b^ Racial and ethnic groups are mutually exclusive. Hispanic persons can be any race.

^c^ Data were compiled from 45 population-based cancer registries that participate in the National Program of Cancer registries, meet the data-quality standards for inclusion in U.S. Cancer Statistics, and meet the criteria for inclusion in the survival data set, which covers approximately 94% of the U.S. population.

Females living in metropolitan areas had the highest one- and five-year (82.2% and 68.8%) relative survival compared to males living in metropolitan areas and to males and females living in non-metropolitan counties.

**Fig 1:**
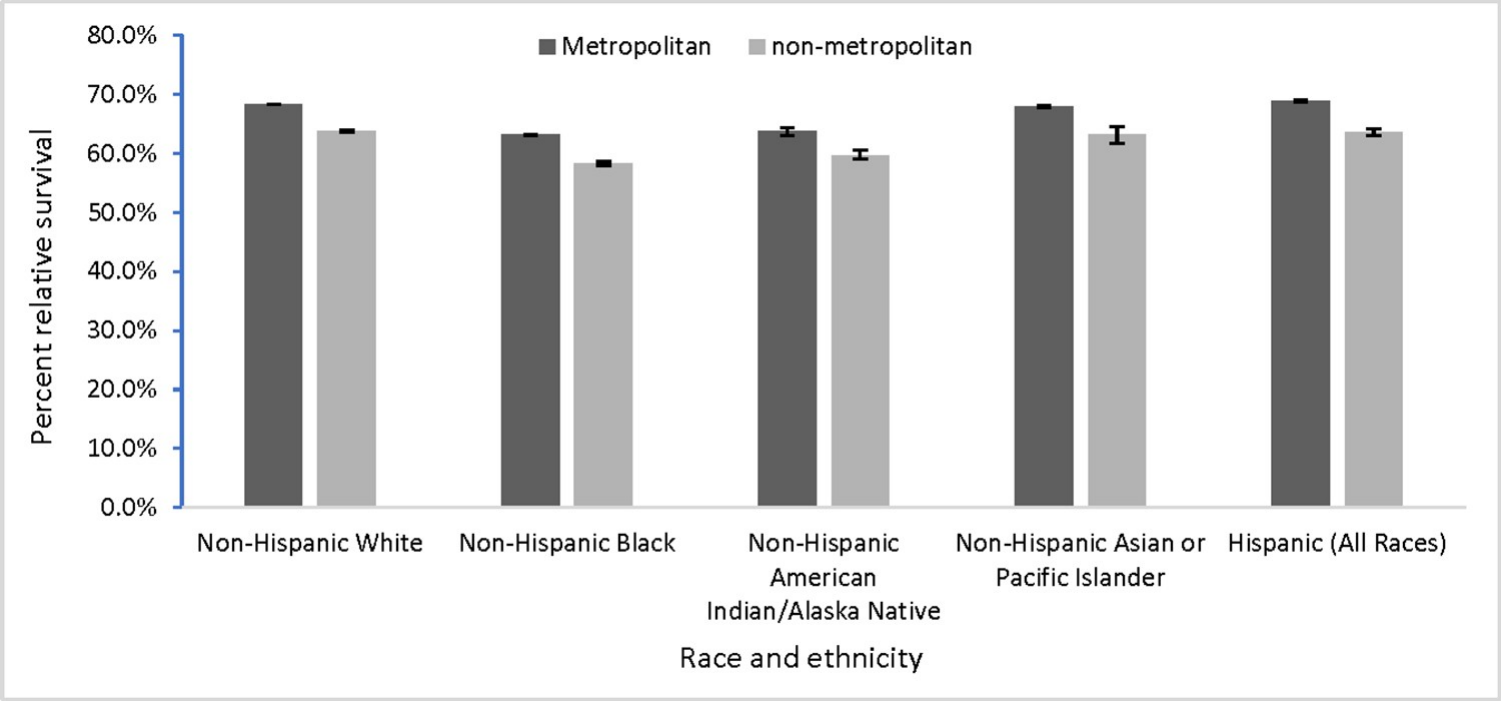
Five-year relative cancer survival by metropolitan and non-metropolitan status^a^ by race-ethnicity United States, 2007–2016^c^. This displays five-year relative cancer survival by metropolitan and non-metropolitan status by race/ethnicity in the United States during 2007–2016. ^a^ The U.S. Department of Agriculture Economic Research Service 2013 vintage rural-urban continuum classification scheme was used to categorize county of residence at diagnosis as metropolitan (rural-urban continuum codes 1–3) or non-metropolitan (rural-urban continuum codes 4–9). ^b^ Racial and ethnic groups are mutually exclusive. Hispanic persons can be any race. ^c^ Data were compiled from 45 population-based cancer registries that participate in the National Program of Cancer registries, meet the data-quality standards for inclusion in U.S. Cancer Statistics, and meet the criteria for inclusion in the survival data set, which covers approximately 94% of the U.S. population. ^d^ Error bars show 95% CIs.

By race and ethnicity, one-year relative survival was highest among non-Hispanic Asian or Pacific Islander persons living in metropolitan counties (82.6%) and lowest among non-Hispanic Black persons (75.4%) and non-Hispanic American Indian/Alaska Native persons (75.4%) living in non-metropolitan counties (Fig 1).

Persons aged 50 years or younger living in metropolitan counites had the highest one- and five-year relative survival (92.2% and 81.5%) compared to persons of other age groups living in metropolitan and non-metropolitan counites.

Compared to persons living in metropolitan counties, those who lived in non-metropolitan counties had lower five-year relative survival for many common cancers including female breast, prostate, lung, colon and rectum, melanoma, urinary bladder, non-Hodgkin lymphoma, kidney and renal pelvis, and leukemia but similar relative survival for corpus uterus cancers (Table 3).

**Table 3:** Relative survival for 10 most common cancer sites^a^ by metropolitan and non-metropolitan status^b^- United States, 2007–2016^c^.

		Metropolitan				non-Metropolitan			
**Cancer site**		*n*	Relative	95% lower	95% upper	*n*	Relative	95% lower	95% upper
Female breast	One-year	2,324,789	97.4%	97.4%	97.4%	404,040	96.8%	96.7%	96.9%
	Five-year	2,324,789	89.8%	89.7%	89.8%	404,040	88.3%	88.2%	88.5%
Prostate	One-year	1,479,825	99.4%	99.4%	99.4%	278,975	99.2%	99.1%	99.3%
	Five-year	1,479,825	98.9%	98.9%	99.0%	278,975	98.3%	98.1%	98.5%
Lung and bronchus	One-year	1,941,581	45.6%	45.5%	45.7%	462,844	42.5%	42.4%	42.7%
	Five-year	1,941,581	19.5%	19.5%	19.6%	462,844	16.4%	16.3%	16.6%
Colon and rectum	One-year	1,438,097	83.3%	83.2%	83.4%	311,669	82.1%	82.0%	82.3%
	Five-year	1,438,097	65.2%	65.1%	65.3%	311,669	63.6%	63.4%	63.9%
Corpus and uterus	One-year	337,429	92.4%	92.3%	92.5%	61,848	92.2%	92.0%	92.5%
	Five-year	337,429	82.4%	82.2%	82.6%	61,848	82.3%	81.8%	82.7%
Melanoma of the skin	One-year	633,786	97.1%	97.0%	97.1%	118,247	96.2%	96.0%	96.3%
	Five-year	633,786	91.4%	91.3%	91.5%	118,247	88.9%	88.6%	89.2%
Urinary bladder	One-year	642,465	89.5%	89.5%	89.6%	134,488	88.9%	88.7%	89.1%
	Five-year	642,465	78.2%	78.0%	78.4%	134,488	76.2%	75.9%	76.6%
non-Hodgkin lymphoma	One-year	643,977	81.9%	81.8%	82.0%	120,517	80.3%	80.1%	80.5%
	Five-year	643,977	71.0%	70.9%	71.2%	120,517	68.1%	67.7%	68.4%
Kidney and renal pelvis	One-year	343,537	87.1%	87.0%	87.2%	70,863	85.0%	84.7%	85.3%
	Five-year	343,537	76.4%	76.2%	76.6%	70,863	72.9%	72.5%	73.4%
Leukemia	One-year	287,323	77.6%	77.4%	77.7%	54,806	74.7%	74.3%	75.1%
	Five-year	287,323	63.4%	63.1%	63.6%	54,806	60.5%	59.9%	61.0%

^a^ Top 10 cancer sites according to rates of new cancer cases in the United States in 2017.

^b^ The U.S. Department of Agriculture Economic Research Service 2013 vintage rural-urban continuum classification scheme was used to categorize county of residence at diagnosis as metropolitan (rural-urban continuum codes 1–3) or non-metropolitan (rural-urban continuum codes 4–9).

^c^ Data were compiled from 45 population-based cancer registries that participate in the National Program of Cancer registries, meet the data-quality standards for inclusion in U.S. Cancer Statistics, and meet the criteria for inclusion in the survival data set, which covers approximately 94% of the U.S. population.

## Discussion

This study used the largest population database available to examine one-year and five-year relative survival among persons diagnosed with cancer in the United States during 2007–2016. It found that females diagnosed with any cancer had a higher one-year and five-year relative survival compared to males. During 2012 to 2016, cancer incidence rates were stable among males and increased among females [1]; whereas, death rates among both males and females decreased [1]. For cancers that affect both men and women, such as lung and colorectal cancer, women were more likely to be diagnosed at an earlier stage compared to men [20]. Studies have found persons diagnosed at an earlier stage have a higher survival [21–23]. Additionally, the types of cancers that comprise “all cancers combined” differ between males and females; historically, a higher proportion of cancers among males are linked with smoking or occupational exposures, these cancers tend to have lower survival [24].

We found one-year relative survival was highest among non-Hispanic Asian or Pacific Islander persons and five-year relative survival was highest among Hispanic persons. One-year and five-year relative survival was lowest among non-Hispanic AI/AN and Black persons. Differences in survival among racial and ethnic groups may be explained by access to high-quality care. Self-reported mammography use from the Behavioral Risk Factor Surveillance System (BRFSS) has typically shown less use of mammography among AI/AN women [25, 26]. Per capita funding for the Indian Health Service, the primary system through which many AI/AN persons access their health care, was $3099 in 2015 compared to $8097 for the US general population [27]. Lack of funding and access to care may contribute to lower relative survival we observed among non-Hispanic AI/AN persons.

Previous studies have examined the differences in cancer death rates among White and Black populations and have found it has narrowed for all cancers combined in men and women [28]. However, the racial disparity remains unchanged for colon and rectal cancer in men, cancers for which incidence and mortality are largely influenced by being screened and access to screening and treatment [29]. Several studies have indicated similar survival by race among patients who receive the same cancer care in clinical trials, suggesting that differences in receiving cancer care may be responsible for racial disparities in survival [29, 30].These differences may include being more likely to be diagnosed at advanced stage of cancer; less likely to be receive standard treatment; and longer delays in diagnosis, follow-up, and treatment [31].

Cancer screening can potentially lengthen the survival interval due to lead time bias, as well as identify relatively slow-growing cancers that have good prognoses [32]. When examining the difference in treatment for leading cancer sites, Zhang et al. [31] found that Black patients were less likely to receive treatment in all cancers except esophageal cancer possibly due to a shortage of medical centers. Lack of access to existing medical centers due to geographic or financial barriers may also contribute, in part, to these disparities. Addressing health disparities caused by differing access to screening and treatment availability may help increase survival among Black cancer patients compared to other racial and ethnic groups.

Relative survival was highest among younger persons. As with sex, this could be due to the mix of cancers diagnosed among younger patients. Additionally, older persons who are diagnosed with cancer often suffer from comorbid chronic conditions and geriatric syndromes which can contribute to the complex treatment decision process [33]. The evidence base for treating older persons is sparse due to underrepresentation in clinical trials, and trials focusing on treatment in older persons are rare [34]. Underrepresentation of older persons in cancer clinical trials means that clinicians have less evidence on how to treat these patients [35]. The American Society of Clinical Oncology (ASCO) has made recommendations for improving the evidence base for treating older adults with cancer which include using clinical trials, leveraging research designs and infrastructure, increasing the authority of the US Food and Drug Administration to incentivize and require research, increasing clinician recruitment of older adults, and using journal policies to incentivize researchers to consistently report on the age distribution and health risk profiles of research participants [35].

We found that persons who lived in non-metropolitan counties had lower survival than those who lived in metropolitan counties, and this difference persisted across sex, race and ethnicity, age, and most cancer types. A recent study showed that rural patients enrolled in clinical trials had similar survival as those who lived in metropolitan areas, suggesting that improving access to high quality treatment may decrease disparities [36]. However, cancer patients living in non-metropolitan areas may not have the same access to these clinical trials. Cancer patients living in non-metropolitan areas often face substantial barriers to receiving optimal treatment, including availability of cancer care providers, distance to services, lack of public transportation, financial barriers, and limited access to clinical trials [37]. Cancer patients living in non-metropolitan areas often have to travel longer distances to get cancer care, such as diagnostic testing, follow-up, and treatment, and may delay or skip getting the care they need. In addition to closures of many rural hospitals, rural hospital or clinics may have fewer resources such as auxiliary or specialty staff and state-of-the-art medical equipment [38].

An ASCO study estimated that 11.6% of oncologists practice in non-metropolitan areas [39]. Improving access to preventive care and treatment for individuals living in non-metropolitan areas may increase survival for individuals diagnosed with cancer in non-metropolitan areas. Residents of non-metropolitan areas also experience comorbid chronic conditions as frequently, if not more, than their urban counterparts [40]. Data from the National Health Interview Survey indicate that cancer survivors in rural areas are more likely to smoke and less likely to be physically active than those in metropolitan areas, which may lead to poorer cancer outcomes [41–43]. Programs that promote smoking cessation, physical activity, and other healthy behaviors may improve survival.

Findings of this study are subject to at least three limitations. First, a higher relative survival might not equate to a lower mortality rate [44]. Results of this study show the percentages of persons who did not die from their cancer within one and five years after cancer diagnosis. Persons with a cancer diagnosis may have died from other causes. Second, although increasing survival over time reflects progress in treating many cancer types, survival trends for some cancers may be due to biases related to screening and early detection [32]. As aforementioned, cancer screening can potentially lengthen the survival interval due to lead time bias [32]. Third, there is evidence of the association between socioeconomic deprivation and increased cancer risk [45]. Results should be carefully interpreted.

This analysis provided a high-level overview of contemporary cancer survival in the United States. Further examination of the differences in cancer survival by cancer type or other characteristics might be helpful for identifying potential interventions that could improve long-term outcomes [46]. Programs that target screening and early detection may help increase survival by diagnosing cancers at an early stage and increasing access to treatment. Programs that help cancer survivors access timely cancer treatment and follow-up care, including promoting healthy behaviors, may also improve survival.
